# In-vivo evaluation of molybdenum as bioabsorbable stent candidate

**DOI:** 10.1016/j.bioactmat.2021.11.005

**Published:** 2021-11-18

**Authors:** Malgorzata Sikora-Jasinska, Lea M. Morath, Maria P. Kwesiga, Margaret E. Plank, Alexia L. Nelson, Alexander A. Oliver, Martin L. Bocks, Roger J. Guillory, Jeremy Goldman

**Affiliations:** a Department of Biomedical Engineering, Michigan Technological University, USA; b Department of Biomedical Engineering and Physiology, Mayo Clinic Graduate School of Biomedical Sciences, USA; c Case Western Reserve University School of Medicine, UH Rainbow Babies & Children's Hospital, Cleveland, OH, USA

**Keywords:** Biodegradable stent, Molybdenum, Biocompatibility, Degradation

## Abstract

Biodegradable stents have tremendous theoretical potential as an alternative to bare metal stents and drug-eluting stents for the treatment of obstructive coronary artery disease. Any bioresorbable or biodegradable scaffold material needs to possess optimal mechanical properties and uniform degradation behavior that avoids local and systemic toxicity. Recently, molybdenum (Mo) has been investigated as a potential novel biodegradable material for this purpose. With its proven moderate degradation rate and excellent mechanical properties, Mo may represent an ideal source material for clinical cardiac and vascular applications. The present study was performed to evaluate the mechanical performance of metallic Mo *in vitro* and the biodegradation properties *in vivo*. The results demonstrated favorable mechanical behavior and a uniform degradation profile as desired for a new generation ultra-thin degradable endovascular stent material. Moreover, Mo implants in mouse arteries avoided the typical cellular response that contributes to restenosis. There was minimal neointimal hyperplasia over 6 months, an absence of excessive smooth muscle cell (SMC) proliferation or inflammation near the implant, and avoidance of significant harm to regenerating endothelial cells (EC). Qualitative inspection of kidney sections showed a potentially pathological remodeling of kidney Bowman's capsule and glomeruli, indicative of impaired filtering function and development of kidney disease, although quantifications of these morphological changes were not statistically significant. Together, the results suggest that the products of Mo corrosion may exert beneficial or inert effects on the activities of inflammatory and arterial cells, while exerting potentially toxic effects in the kidneys that warrant further investigation.

## Highlights

• Mo implants in mouse arteries avoided neointimal hyperplasia over 6 months.
• Quantification of CD31-labeled arterial sections showed an avoidance of significant harm to regenerating endothelial cells for the Mo implants.
• Qualitative inspection of kidney sections showed a potential pathological remodeling, indicative of possible impaired filtering function.

## Graphical abstract

**Figure:**
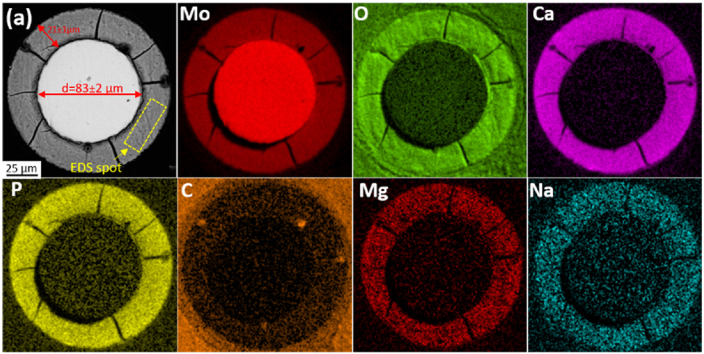

## 1 Introduction

Biodegradable stents or scaffolds (BRS) have been pursued as the potential “holy grail” for the treatment of coronary artery disease and other vascular obstruction for the last two decades. The possibility that a device could re-establish vessel patency, undergo a latent degradation process, and allow for vascular remodeling with the restoration of normal vasomotor function is the reason BRS continue to be investigated and developed. Although polymeric scaffolds were the first to receive FDA approval in 2016 with the Absorb bioresorbable vascular scaffold (Absorb BVS) (Abbott Vascular, Santa Clara, California), subsequent widespread real-world use of Absorb BVS was associated with unacceptably high rates of late target lesion failure and scaffold-associated thrombosis, which led to its withdrawal from the market in 2017 [[1], [2], [3], [4]]. Although polymeric BRS are still being pursued, the limitations experienced by the real-world use of the Abbott's Absorb BVS after tremendous research & development expenditures, gives other companies reason for pause. Furthermore, the goal of making thinner polymeric scaffolds to overcome some of the previously encountered problems will continue to be limited by the inverse relationship of the need to maintain sufficient radial force for a long enough period to allow vascular remodeling.

Metallic BRS are a very promising alternative to polymeric BRS due to their ability to: have excellent radial force with relatively thinner struts, be implanted more uniformly, and have a clear increase in radiopacity. Magnesium, Zinc, Iron (Mg, Zn, Fe), and their respective alloys continue to attract the attention of both industry and the scientific community as promising materials for endovascular stents or scaffolds. By far, the largest number of studies related to the clinical translation of cardiovascular stents involves Mg-based devices. The Biotronik Magmaris (Biotronik AG, Bülach, Switzerland) Mg scaffold received initial CE Mark in June 2016. Despite encouraging clinical trial data, the device remains restricted to use in Europe for low risk de novo lesions and with very specific favorable anatomy [5].Despite the potential advantage of having smaller strut thickness compared to polymeric BRS, the Magmaris magnesium stent still possesses a relatively large strut thickness compared to currently available DES, such as the Xience DES (Abbott Vascular, Santa Clara, California) (150 μm vs. 81 μm) [6,7]. Thicker struts are associated with higher degrees of neointimal hyperplasia in in-stent stenosis, device thrombosis, reduced deliverability, poorer wall apposition, and decreased long-term safety [[8], [9], [10]]. The inability to reduce strut size in a stent represents a serious disadvantage, since stent strut size is inversely related to efficacy and has progressively decreased due to technological innovations [11]. Alternatively, Fe-based materials exhibit superior mechanical properties but their localized corrosion, and voluminous insoluble corrosion products do not allow Fe stents to be uniformly degraded, impacting their mechanical integrity at later stages of dissolution [12]. The development of Zn-based stents has been challenged due to the intrinsic low strength of Zn-based alloys and mechanical instability arising from a strong strain rate sensitivity and strain softening behavior [[13], [14], [15]]. There are no reports on the clinical performance of Zn-based cardiovascular implants. However clinical trials of a sirolimus-eluting Fe stent is in progress [16,17].

Molybdenum (Mo) has emerged as a promising candidate for vascular stent applications, which may surmount the limitations of previously explored Fe, Zn, and Mg alloys [18,19]. As a result of a higher elastic modulus, yield strength, tensile strength, and density relative to the other absorbable metals under development and even relative to permanent materials such as stainless steel and cobalt-chromium, Mo possesses better radio-opacity and radial strength [20,21]. Such properties have enabled stent struts to become thinner while retaining an excellent ability to resist deformation and maintain radial force. Thinner struts are widely recognized as advantageous as they improve flexibility, facilitate re-endothelialization, decrease disruption of shear stress, reduce peri-strut inflammation, and ultimately decrease the degree of vascular injury during implantation [7,[9], [10], [11]]. Clinically, this has corresponded with a decrease in restenosis and improved deliverability. Due to a very high melting point of 2,620 °C, Mo retains its strength and creep resistance even at high temperatures, and it's strength increases even further as the material is cold-worked [20]. In contrast to other metals, the ductility of Mo also increases with increased cold working [20,22,23].

In addition to Mo's exceptional mechanical properties, Mo is also a biologically relevant trace metal. Although Mo has received less attention than other essential microelements for its role in human health, it remains crucial for the function of the human body [24]. Mo is involved and required for multiple enzymatic processes [24,25]. Toxic effects of Mo have been reported in rodents including mild renal failure, reproductive effects, and depression [[26], [27], [28], [29], [30], [31]]. The administration of Mo to mice resulted in Mo accumulation in the liver (18% of the total dose), followed by kidney (9%) and pancreas (3%) [32]. There is limited information to suggest toxicity of Mo to humans [24,33]. Significant amounts of Mo are excreted through both feces and urine, with urine being the primary route of elimination [33]. Furthermore, human studies looking at excessive Mo intake demonstrated the body's capacity to increase urinary excretion in response to elevated plasma levels [34]. Increasing dietary Mo intake from 22 to 72 mg/d resulted in a triple increase of Mo transfer from plasma to urine, and an additional double increase was observed when subjects transitioned from 121 to 467 mg/d [34]. The ability of the body to adapt to a wide range of Mo intake and plasma levels could be the reason that high or low Mo intakes have minimal impact on human health. The upper intake recommended for North America has been set at 2 mg/d [34]. The European Commission has suggested an upper limit of 0.6 mg/d [34].

The therapeutic uses of Mo are developing in several medical sectors. The more commonly known uses of various Mo compounds include the treatment of anemia, prevention of dental caries, and the treatment for Wilson's disease [35,36]. Other potential therapeutic uses are under development as alternatives for treating esophageal cancer with Mo compounds [37]. Another research direction includes study of the therapeutic benefits of Mo organometallic complexes on human leukemic T-cells [37]. While Pure Mo has not yet been investigated *in vivo* as an implant material, there has been an increasing trend over the past two decades in developing Mo containing biomaterials for cardiovascular and orthopedic applications. Co–Cr–Mo and Ti–Mo alloys and wear resistant Mo-based coatings are several examples, although they have little overall molybdenum release due to their overall high stability [[38], [39], [40], [41]].

In the present study, pure Mo was evaluated in detail for its potential as an emerging biomaterial candidate for manufacturing ultra-thin biodegradable stents. The microstructural, mechanical and corrosion properties, as well as the *in vivo* biocompatibility, were systematically investigated.

## 2 Materials & methods

### 2.1 Materials

Annealed high purity 125 μm diameter Mo wire purchased from Goodfellow Corporation (Huntingdon, UK) was used for mechanical testing and implantation in the murine artery. The specimens for the *in vitro* corrosion assessment were cut from hot formed, annealed 14 mm diameter Mo rod purchased from Plansee (Slough, UK).

### 2.2 Microstructure & mechanical properties

The samples were ground and polished following standard metallographic procedures and etched with chemical etchant composed of hydrofluoric acid, nitric acid and lactic acid in the ratio 1:2:6 by volume. Microstructure was recorded by scanning electron microscopy (Philips XL 40 ESEM). The grain size was evaluated according to ASTM E112-96 standard, following the linear intercept procedure. Mechanical properties were evaluated by tensile testing for Yield Strength (YS), ultimate tensile strength (UTS) and fracture elongation (EF). Tensile tests were carried out on wire segments with a gauge length of 40 mm using a 100 lb load cell at a strain rate 1·10^−3^s^−1^_._

### 2.3 In vitro and *in vivo* corrosion evaluation

Open circuit potential, potentiodynamic polarization (ASTM G59–97), electrochemical impedance spectroscopy (EIS) and static immersion tests (ASTM G31-72) were performed to investigate the corrosion behavior of pure Mo. The physiological solution was prepared with 9.5 g of balanced Hanks’ salts (H1387, Sigma-Aldrich) in 1 L of distilled water supplemented with 0.35 g of NaHCO_3_ (S8875-500 G, Sigma Aldrich). The pH of all solutions was measured and adjusted to 7.4 by using 1 M NaOH or HCl aqueous solutions; The solution temperature was adjusted to 37 ± 1 °C during the tests. The sample surfaces were polished with 4000 grit SiC paper and 0.05 μm alumina suspension and then washed with ethanol for 5 min in an ultrasonic bath to remove residuals of abrasive particles, then rinsed with distilled water for 10 min prior to corrosion testing.

The potentiodynamic test was performed using a conventional three-electrode cell (Princeton Applied Research Model K47) with a platinum counter electrode of 1 cm^2^ surface area, a saturated calomel reference electrode and the prepared Mo working electrode. The open circuit potential (OCP) was monitored without applying any outside source for 3600 s until equilibrium was reached at the corrosion potential E_corr_. A scan rate of 0.166 mV/s, with an applied potential range of 1 V, was employed. The experiments were carried out in an aerated environment at 37 ± 1 °C. The solution was stirred with magnetic agitation during the test. For each type of material, four specimens were tested using the same experimental conditions. The corrosion rates were obtained based on the calculated corrosion current density (і_corr_), using the following equation:

(Eq. 1) $$CR=3.27\cdot 10^{-3}\frac{i_{corr}EW}{\rho }$$

where CR is the corrosion rate (mm·year^−1^), i_corr_ is the corrosion current density (μA·cm^−2^) obtained based on potentiodynamic curves using a Tafel extrapolation method, EW is the weight equivalent and ρ is the material density (g cm^−3^). Impedance spectra were separately collected from 100 kHz to 0.01 Hz with 5 mV perturbation amplitude. For static immersion tests, samples were immersed for 336 h using approximately 30 ml of solution per cm^2^ of sample surface. The containers were placed in the incubator (T = 37 ± 1 °C, and a relative humidity of 90%). The whole volume of solution was changed every 48 h to keep the pH value close to 7.4 and to maintain conditions as constant as possible. Subsequently, the samples were washed with 200 g L^−1^ of chromium oxide to remove corrosion products before measuring the final weight. Corrosion rates were calculated using the weight loss method, following the equation:

(Eq. 2) $$CR_{S}=8.74\cdot 10^{4}\frac{W}{A\cdot t\cdot \rho }$$

CR_S_ is the corrosion rate (mm·year^−1^), W is the weight loss (g), A is the exposed area (cm^2^), t is exposure time (h) and ρ is the material density (g·cm^−3^). Four specimens were tested for each condition and used to calculate average values and relative standard deviations. Fourier-transform infrared spectroscopy (FTIR) was conducted in diffuse reflectance mode with a Jasco FTIR-4200 spectrophotometer. A series of 64 scans were performed at 4 cm^−1^ resolutions from 400 to 4000 cm^−1^ to evaluate the chemical composition of the Mo samples after static immersion tests.

Mo explants from mouse arteries were stored in absolute ethanol at room temperature and then placed in a desiccator until the remaining biological material dried. Each wire sample was affixed to a plastic mounting clip and embedded in epoxy resin. All samples were then mechanically polished following standard metallographical procedures. The surface morphologies and chemical compositions of the *in vitro* and *in vivo* corroded samples, both before and after the corrosion testing, were examined by SEM equipped with a tungsten filament and operated with an acceleration voltage of 15 kV. Micrographs were acquired with a probe current in the range 1·10^−10^ - 1·10^−8^ mA. The explant diameter and the thickness of the corrosion film was calculated based on the SEM micrographs of 20 Mo wire sections. All quoted errors correspond to the sample standard error. In addition, X-ray diffraction (XRD) was performed on the explant covered by degradation products (XDS2000 θ/θ X-ray diffractometer, Scintag Inc., Cupertino, CA with CuKα radiation (λ = 1.540562 Å). The scans were performed continuously from 10° to 60° in 2θ at a speed of 0.2°/min with a step size of 0.02°.

### 2.4 In vivo biocompatibility evaluation

Mo and platinum (Pt) (bioinert control material) wires were cut into 1 cm long segments and sanitized in 70% ethanol before being surgically implanted into live mouse arteries (n = 16 for Mo and n = 12 for Pt implants). Each wire was implanted into the abdominal aorta mouse lumen following adaptation of the surgical protocol reported previously [42]. Briefly, the wall of the abdominal aorta was penetrated by the sharpened wire, which was then advanced near the arterial endothelium for ∼7 mm before being punctured out of the artery, leaving approximately 5 mm of the metal wire within the arterial lumen. The implant was placed in the aorta at a location distal to the renal bifurcation. The animal study was approved by the Michigan Technological University Institutional Animal Care and Use Committee (IACUC) and was performed in accordance with the Panel on Euthanasia of the American Veterinary Medical Association. At 6 months post-implantation, mice were euthanized and arteries containing the degraded wires were removed for subsequent histological and metallographic analysis. The extracted wire samples intended for histological analysis (n = 9 per group) were snap-frozen in liquid nitrogen and cryo-sectioned [43]. The remaining wires were used for material characterization. Prior to analysis, samples were preserved in a −80 °C freezer.

Cross sections were fixed with neutral buffered formalin solution and then stained with hematoxylin and eosin (H&E) and mounted in Eukitt® Quick-hardening mounting medium (03989, Sigma-Aldrich). Sections were also fluorescently labeled against CD31 (for endothelial cell evaluation-ab28364, abcam) or alpha actin (for smooth muscle cell evaluation - ab5694, abcam) following paraformaldehyde and methanol fixation, respectively. The sections were also stained with DAPI and mounted with Fluoromount aqueous mounting medium (F4680, Sigma-Aldrich). All stained specimens were imaged using a Zeiss AxioScan.Z1.

A different AxioScan.Z1 profile was optimized for each fluorescent stain (CD31, *⍺-*SMA, mcherry). Extended depth of focus was performed on the z-series obtained. The best fit function was applied to the histogram of pixel intensities in the CD31 images. The min/max function was used for the histogram of pixel intensities on the *⍺*-SMA images, resulting in the following numbers for each sample type: Mo - black at 0, white at 64045, Pt - black at 159, white at 35730. Mcherry images were taken as produced by the AxioScan without alteration.

Adult transgenic mice were used as host subjects to evaluate the stent metals [44]. In this mouse line, a fluorescent mCherry reporter is fused to the human diphtheria toxin receptor, under the control of the genetic promoters LysM and Csf1r. This transgenic mouse specifically labels monocyte/macrophage populations, but not dendritic cells, with the red fluorescent protein derivative mCherry. Since monocyte/macrophage populations become red fluorescent in this mouse line, the evaluation of inflammation against the stent metal implants is dramatically simplified.

The kidneys from mice in each implant group (n = 6 for both Pt and Mo) were used to measure the area of the Bowman's capsules and glomeruli. Longitudinal 10–12 μm sections for each kidney were prepared with a cryostat (HM525NX, Thermoscientific) and placed on Histobond slides. The cross sections were stained with hematoxylin and eosin (H&E), preserved in mounting medium, and imaged. A total of 40 capsule area measurements were made from two different kidney locations of each mouse by random selection of capsules. The ratio of glomerulus area to Bowman's capsule (abbreviated as *G/B*) was also obtained. Images used for a qualitative analysis were obtained with an Olympus microscope (BX51). A two-sample *t*-Test was performed on the data and a p-value < 0.05 determined statistical significance.

### 2.5 Statistical analysis

The experimental results are presented as the mean ± standard deviation unless otherwise noted. The standard deviation was based on four experiments in the case of mechanical testing and corrosion experiments. The H&E stain was used to measure the neointimal area (NA) and wire lumen thickness (WLT), metrics that have been described in detail by Oliver et al. [45]. The measurements were analyzed for significant differences in the mean with a Wilcoxon rank sum test and variability with an F-test. P-values less than 0.05 were considered significant. Immunofluorescence measurements were assessed for normality and normally distributed immunofluorescent data was evaluated with a parametric student's t test, while non normally distributed data was evaluated with a non parametric wilcoxon rank sum test.

## 3 Results & discussion

### 3.1 Microstructure and mechanical properties

**Fig. 1:**
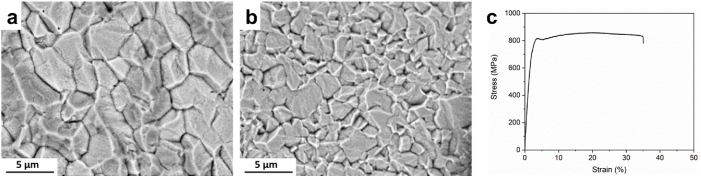
Microstructure of the pure molybdenum a) rod, b) wire and c) mechanical properties of Mo wire.

Fig. 1a and b show SEM images of the Mo rod and wire, respectively. Both materials with different processing histories represent reasonably equiaxed grain structure with average grain size of 2.4 ± 0.5 μm and 0.9 ± 0.1 μm for extruded rod, and wire, respectively.

However, the wire features a significantly smaller grain size, which is attributed to plastic deformation introduced by wire drawing. Indeed, severe cold deformation followed by annealing results in remarkably fine structures in metallic materials. It is worth noting that the equiaxed microstructure provides an isotropic mechanical performance and is therefore of paramount importance for biomedical implants that are subjected to multidirectional cyclic loading. Fig. 1c depicts the tensile stress-strain curve for the Mo wire at an initial strain rate of 1·10^−3^s^−1^. As demonstrated in the figure, the Mo wire possessed a YS of 673 ± 12 MPa, a UTS of 868 ± 7 MPa, and a EF of 35 ± 4%. Similarly, the annealed Mo rod exhibited high mechanical strength (YS = 515 MPa, UTS = 620 MPa as specified by Plansee, Slough, UK). In contrast, the fracture elongation of the rod is significantly lower than for the wire (18% vs 35%), implying that the reduction in Mo grain size leads to increased tensile ductility [20,22,23]. Thus, the measured tensile properties of the Mo wire overly exceed the mechanical benchmarks for an ideal degradable stent material to such an extent that no reported pure metals have exhibited such high mechanical properties.

#### 3.1.1 In vitro corrosion of molybdenum

**Fig. 2:**
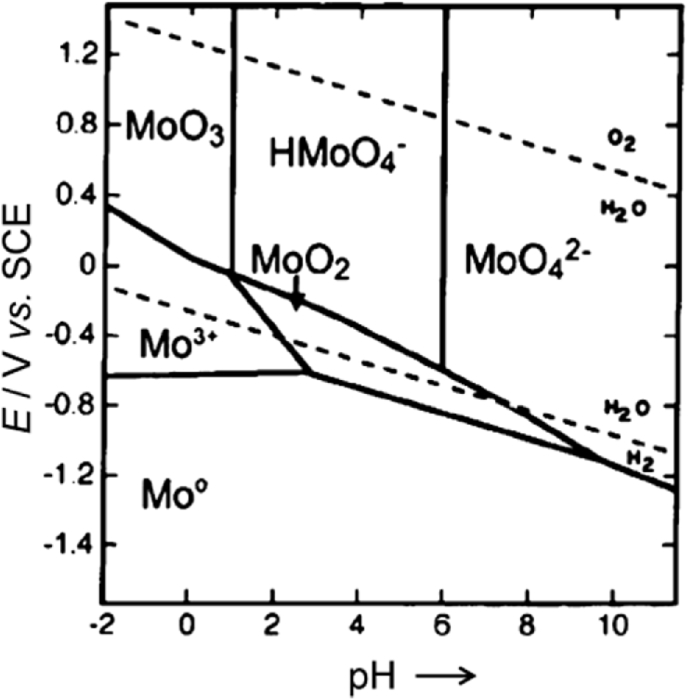
Potential-pH diagram of a Mo–H_2_O system [46].

Despite several studies on Mo, knowledge related to its corrosion mechanism is incomplete. Mo is a relatively base metal, similar to Fe, with its domain of thermodynamic stability lying completely below that of water, as shown in Fig. 2 [46]. Mo has a partial domain of passivity associated with the formation of MoO_2_ across the whole pH range, but is unstable to oxidation from the Mo^IV^ to the Mo^VI^ state (transpassive) at higher potentials [47]. Thus, Mo dissolves at more positive potentials at 7.4 pH according to the reactions [18,48]:

(Eq. 3) $$Mo+4H_{2}O\to MoO_{4}^{2-}+8H^{+}+6e^{-}$$

(Eq. 4) $$Mo+2H_{2}O\to MoO_{2}+4H^{+}+4e^{-}$$

(Eq. 5) $$MoO_{2}+2H_{2}O\to MoO_{4}^{2-}+4H^{+}+2e^{-}$$

In the aerated environment, the Mo dissolution is accompanied by oxygen reduction according to the following reaction [47]:

(Eq. 6) $$O_{2}+4e^{-}+\;2H_{2}O\to 4OH^{-}$$

The Pourbaix diagram for Mo (Fig. 2) shows that in an acidic environment (i.e. urine), Mo tends to be passivated, while under neutral or slightly alkaline conditions (i.e. blood plasma) no surface oxides are stable [46]. The passivation in such an environment is not effective due to the formation of soluble species involving HMoO_4_, MoO_4_. The major corrosion products in an alkaline environment were identified as Mo^III^ oxide/hydroxide, and MoO_2_ [48,49]. Consequently, in neutral-to-alkaline conditions, where Mo oxyanions become more stable, dissolution of Mo increases. Oxidizing conditions in the physiological environment (caused by inflammatory reaction) might severely increase Mo corrosion susceptibility resulting in higher degradation rates. The *in vitro* and *in vivo* degradation evaluation revealed the presence of several elements such as Mo, P, O, Ca, Mg, and Na among the corrosion products, suggesting the formation of oxides, hydroxides and molybdates. This is a result of the ionic interaction between tetrahedral $MoO_{4}^{2-}$ oxyanions reacting with magnesium, calcium, sodium, and phosphate ions which are components of Hanks’ solution and blood plasma.

**Fig. 3:**
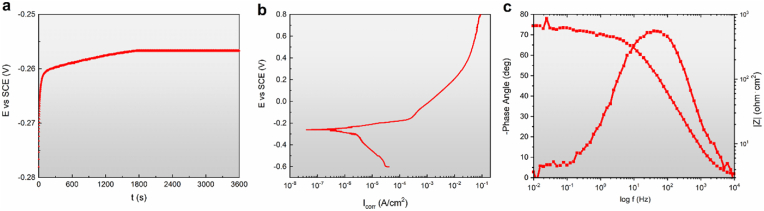
Electrochemical measurement plots for pure Mo in Hanks' solution a) OCP, b) potentiodynamic polarization curves, and c) EIS bode plots.

The representative electrochemical measurement plots for pure Mo are presented in Fig. 3*.* In buffered Hanks’ solution the open circuit potential increases sharply after the first minutes of immersion and reaches a value of −260 mV after 1 h (Fig. 3a). The value of the corrosion potential (E_corr_) is 265 mV. The Mo polarization curve (Fig. 3b) contains a well pronounced Tafel region. Therefore, E_corr_ and corrosion current density (i_corr_) could be easily determined by Tafel extrapolation. The corrosion rate calculated based on electrochemical measurements is 4.2 ± 0.21 μm/year. The value of the corrosion rate based on the short-term weight loss tests (3.92 ± 0.18 μm/year) coincided with electrochemical measurements. The EIS spectra (Fig. 3 c) show that in the higher frequency region, log∣Z∣ tends to become constant, with the phase angle values falling rapidly towards 0° with increasing frequency. This is a response typical of resistive behavior and corresponds to the solution resistance, R_Ω_. In the medium frequency range, a linear relationship between log∣Z∣ and log f is observed and phase angle maxima (less than −90°), indicating that the protective film on Mo was not fully capacitive after 1 h immersion time.

**Fig. 4:**
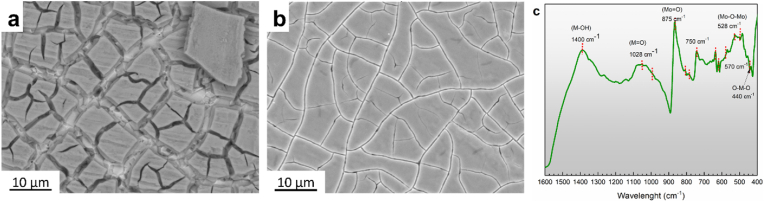
(a) & (b) SEM images of samples' surface morphology at different spots after static immersion test in Hanks' solution for 14 days, (c) representative FTIR pattern of Mo sample after 14 days immersion in Hanks' solution.

The 14-days immersion test resulted in the formation of compact and homogenous shell-like structures containing Mo, O, Ca, Ma, Na, and P, as seen in Fig. 4a and b. Fig. 4c shows the FTIR spectra of Mo samples degraded in Hanks’ solution acquired in the wave number region between 1600 cm^−1^ and 400 cm^−1^. The results of the analysis suggest the formation of a mixture of Mo-based compounds during the dissolution of the metal in the physiological environment. The chemistry of Mo in its higher oxidation states is dominated by molybdate compounds (such a as calcium molybdate (such as CaMoO_4_, MgMoO_4_ and/or Na_2_MoO_4_) [[49], [50], [51]]. The strong absorption bands situated at 750-875 cm^−1^ are originated from the antisymmetric stretching vibrations in the tetrahedral MoO_4_^2-^ [50,52]. The splitting of the Mo=O stretching at 993 and 1028 cm^−1^ reflects the good crystallinity of the protective films. The bending infrared modes are observed in the lower frequency region (400–500 cm^−1^). The absorptions around 1400 reveal the vibration of the Mo–OH bond [49]. These findings are consistent with reported dissolution surface chemistry of bulk Mo materials in neutral and slightly alkaline aqueous solutions.

### 3.2 In vivo corrosion progression

**Fig. 5:**
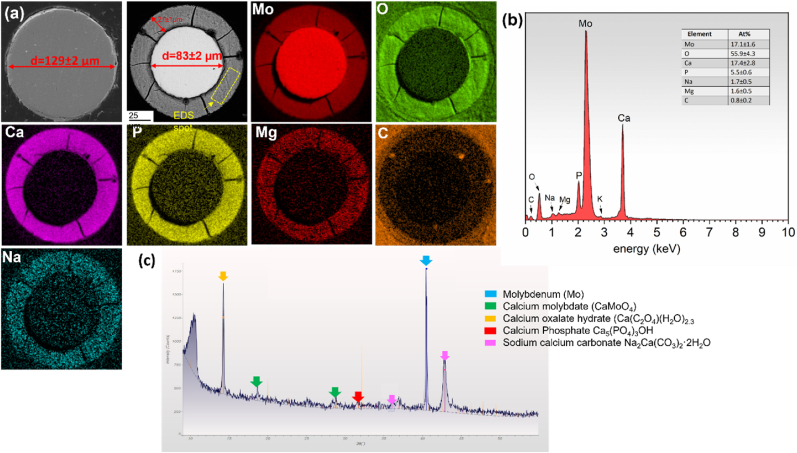
a) As received Mo wire cross section, and Energy dispersive X-ray maps of representative Mo explant cross-sections, b) quantitative EDS analysis of the protective film formed on the surface of the wire, c) XRD on the explant.

Mo wires were observed to undergo progressive degradation while implanted *in vivo* in the murine artery (Fig. 5). The initial diameter of the Mo implant was 128.9 ± 2.4 μm. The area of metallic Mo decreased by 34.5 ± 1.5% after six months of *in vivo* implantation, leaving behind an 85.2 ± 2.6 μm diameter wire surrounded by a 21.2 ± 1.8 μm thick corrosion film. Corrosion appeared to primarily occur uniformly on the surface of the wires. Degraded metallic Mo was replaced by a homogenous layer of corrosion product as implantation time progressed. The corrosion products formed within the nominal cross-sectional area of the wire and the implant retained its original shape. EDS analyses confirmed the presence of a large amount of Mo, O, Ca, and P, along with Na and Mg in the corrosion film (Fig. 5). Surprisingly, in contrast to the other biodegradable metallic materials, the protective film that formed on Mo did not reveal a layered structure. All detected elements appeared to be uniformly distributed through the degradation film, as seen in Fig. 5a. XRD on the explant surface further confirmed the presence of the compounds including calcium molybdate, calcium oxalate, calcium phosphate and sodium calcium carbonate (Fig. 5 c).

### 3.3 In vivo biocompatibility response to pure molybdenum

**Fig. 6:**
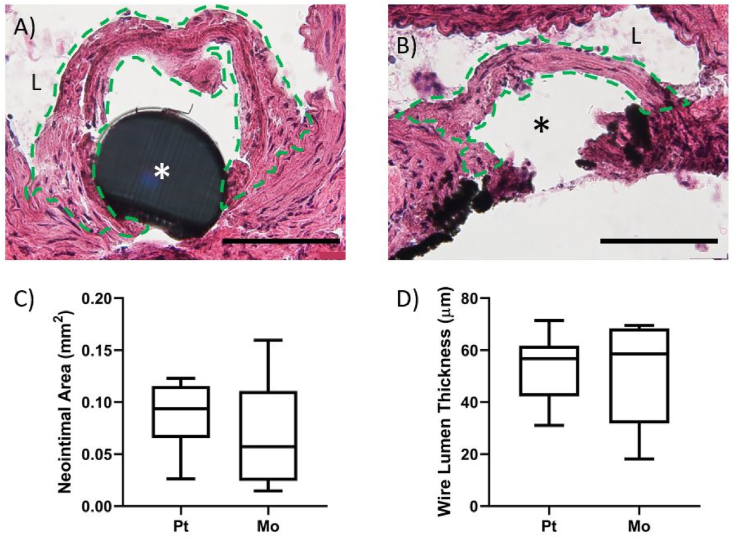
a) Platinum and b) molybdenum cross-sections stained with hematoxylin and eosin and imaged at 600x normal magnification. The scale bars equal 100 μm. The area outlined in green identifies the neointimal tissue that formed around the implants, the “L” indicates the arterial lumen, and the “*” indicates the implant location. Please note that the arterial wall adjacent to the Mo implant was damaged during cross-sectioning due to the very hard Mo wire being pushed through the soft biological tissue during the cryo-sectioning process, which did not occur for the platinum wire-implanted control tissue. This was an unavoidable processing defect that left the neointimal tissue unharmed and did not alter the neointimal measurements. C) and d) compare neointimal area and wire lumen thickness, respectively, between Pt and Mo. Sample size for the measurements was n = 9 for Mo and n = 8 for Pt groups. (For interpretation of the references to colour in this figure legend, the reader is referred to the Web version of this article.)

When examining the *in vivo* cross-sections, the neointimas formed around biostable Pt control implants appear to be qualitatively larger than that of Mo (Fig. 6a & b). Quantitative analyses shown in Fig. 6c and d demonstrate that there is no significant difference between Mo and Pt for neointimal area (NA) or wire-to-lumen thickness (WLT) metrics.

**Fig. 7:**
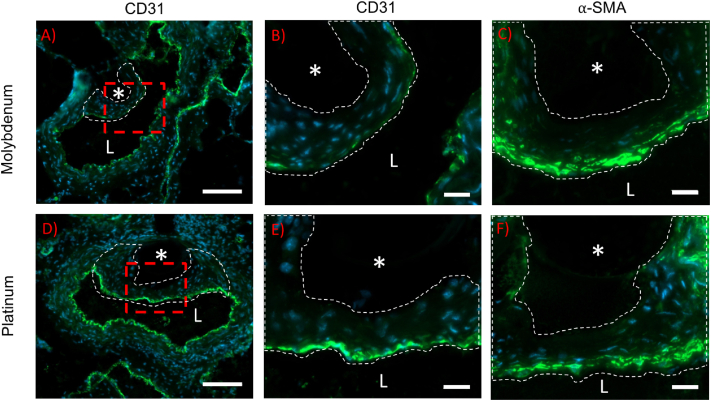
CD31 and ⍺-SMA immunofluorescence staining of arterial cross sections containing molybdenum (A, B, and C) and platinum (D, E, and F) implants. Images in B and E represent magnified neointimal regions of the red dashed box outlined in images A and D, respectively. In each image, the white dashed line outlines the neointima, “L” identifies the arterial lumen, and “*” identifies the wire implant location. CD31 labeling (green) is shown in A, B, D, and E. Smooth muscle alpha actin labeling (green) is shown in images C and F, for different specimens. All cell nuclei are stained with DAPI (blue). Images were taken at 400x normal magnification, z-stacked, and extended depth of focus was performed. The scale bar for A and D is 100 μm, and for B, C, E, and F is 20 μm. (For interpretation of the references to colour in this figure legend, the reader is referred to the Web version of this article.)

Due to the endothelial cell (EC) layer's importance for avoiding thrombotic events after stent deployment, EC regeneration was inspected in Pt and Mo arterial cross sections by CD31 labeling (Fig. 7a, b, d, e). Although a stable neointima forms around both implants without signs of thrombus, the CD31 positive signal appears more intense and uniform in the Pt sample. However, the reduction in CD31 signal intensity and coverage for the Mo group relative to Pt is not statistically significant (Table 1). Interestingly, there was a general reduction in CD31 signal intensity from the native endothelium to the neointimal endothelium, which was highly significant for the Pt implant group (Table 1). This suggests that CD31 expression may reduce in the regenerated endothelium relative to the native endothelium. Because smooth muscle cells (SMCs) typically predominate within the neointimal tissue of arterial implants and contribute to arterial failure, *⍺-*SMA labeling was used to inspect SMC presence and distribution within the neointima of the implants (Fig. 7c & f). The Pt and Mo samples demonstrate a similar modest level of SMC neointimal content (Table 1), with a greater signal intensity and cellular organization on the luminal side of the neointima vs. the mural interface for both implant materials.

**Table 1:** Immunofluorescence measurements and statistics.

	Mo	Pt	p values w/description	sample size Mo	sample size Pt	comments
CD31 average intensity at neointima	8.5 ± 2.4	10.1 ± 3.6	*0.2571,*Mo vs Pt	4	6	numbers are normalized to dapi intensity (sum/sum)
CD31 average intensity at native endothelium	13.1 ± 4.6	18.8 ± 5.3	Pt: *0.0087, Pt native vs Pt neointima Mo: 0.0733, Mo native vs Mo neointima	4	6	numbers are normalized to dapi intensity (sum/sum)
CD31% coverage of neointima	55.9 ± 13.0%	67.5 ± 19.3%	0.2885, Mo vs Pt	4	6	
Alpha actin % coverage	4.0 ± 2.8%	5.9 ± 6.3%	0.4801, Mo vs Pt	6	7	numbers are normalized to area

Immunofluorescence measurement data and statistical details for CD31 and alpha actin labeling. All data was compared by ttest except for the italicized p values, which were tested by rank sum.

Due to the importance of inflammatory cells as mediators of restenosis of arterial implanted materials, the endogenous mcherry fluorescence was imaged in tissue cross sections. Only a few cells were mcherry positive per tissue section and these were generally randomly distributed throughout the cross section, suggesting a very low chronic inflammation activation against either Pt or Mo arterial implants in the mice. The findings with the mcherry imaging were confirmed with CD68 macrophage labeling (data not shown).

Corrosion byproducts that enter the blood are filtered through the kidneys prior to urinary excretion. Therefore, an evaluation of the kidneys may indicate systemic corrosion product toxicity. A pathological remodeling of kidney Bowman's capsule and glomeruli is indicative of impaired filtering function and development of kidney disease [[53], [54], [55]]. A qualitative analysis revealed collapsing glomerular tufts and distinct hypercellularity of the glomeruli in the Mo compared to the Pt group (Fig. 9a & 9b**)**. Other noticeable changes included localized areas of expanded mesangial matrix. However, we found no significant difference in the area of the Bowman's capsules or glomerulus from mice implanted with Mo compared to Pt. Bowman's capsule area decreased non-significantly from 4800 ± 860 μm^2^ (Pt) to 4700 ± 690 μm^2^ (Mo), (p = 0.80, Fig. 9). Glomerulus area also decreased non-significantly from 3700 ± 600 μm^2^ (Pt) to 3460 ± 370 μm^2^ (Mo), (p = 0.45, Fig. 9). There was also no statistically significant difference in the *G/B* area ratio between the Pt and Mo groups (0.77 ± 0.04 and 0.73 ± 0.04, respectively).

The urinary excretion of Mo is in the range of 17–80% of a total dose ingested [33]. Bompart et al. [28] showed that sub-chronic exposure to low doses (40 mg/kg/d) of Mo had unremarkable effects in rats. On the contrary, higher doses (80 mg/kg/d) in the same study resulted in kidney injury, which was demonstrated by a decrease in glomerular filtration rate, in conjunction with increases in alanine aminopeptidase, glutamyl transpeptidase and urinary kallikrein, which are indicative of glomeruli and tubular dysfunction.

Since a biodegradable cardiovascular stent service lifetime is approximately 2 years [45], the persistent kidney exposure to Mo degradation products could lead to negative renal effects. A morphometric analysis of capsule and glomerulus can be used to assess kidney function and disease [[55], [56], [57], [58]]. Sasaki et al. [55] proposed that changes in glomerulus capillary to Bowman capsule volume could be important in assessing filtration in the kidneys. In their study, an average ratio of 0.63 ± 0.05 was found in kidney biopsy samples obtained from human donors with no obvious signs of renal disease. Another study related the *G/B* ratio to the development of glomerular hypertrophy in diabetic mice [56]. The reported ratios ranged from approximately 73%–81% in diabetic mice compared to 63%–67% in normal mice from 4 to 12 weeks, demonstrating that G/B ratios increase under disease conditions. In our study, the G/B ratios slightly decreased, but not significantly. Our preliminary work on kidney sections (presented in Fig. 9) demonstrates the absence of quantitative effects on the kidneys. However, qualitative changes were evident, in terms of cellularity and shape of the glomerulus due to Mo wire implantation in the abdominal aorta.

This raises concerns regarding the long-term systemic effects of Mo. Moreover, a number of other studies have reported Mo-dependent alterations in kidney function and structure [33,[59], [60], [61], [62]]. Based on our findings with Mo arterial implants, additional investigations into renal function in response to Mo arterial implants are warranted, such as measurements of blood urea nitrogen, as well as creatinine and proteinuria levels. When considering the origin of the possible systemic toxicity of pure Mo wires degrading in-vivo, the pourbaix diagrams allude to the molybdate anion $MoO_{4}^{2-}$ as the dominating soluble corrosion product. Molybdate indeed possesses a key enzymatic functionality, serving as a cofactor for a variety of enzymes such as sulphite and xanthine oxidoreductases [24]. It has recently been shown *in vitro* that supraphysiological molybdate supplementation can inhibit protein tyrosine phosphotases at 1 mM and increase reactive oxygen species production at 100 μM in liver and kidney cells [63]. It is unclear whether chronic delivery of the molybdate anion from the circulation via degradation processes *in vivo* is sufficient to elicit similar effects. Since the G/B ratio can be influenced by age, sex, and overall health conditions in rodents, a properly designed toxicological study of implant derived degradation products and nephrotoxicity should be implemented to determine whether the qualitative histological remodeling seen here is harmful or benign.

**Fig. 8:**
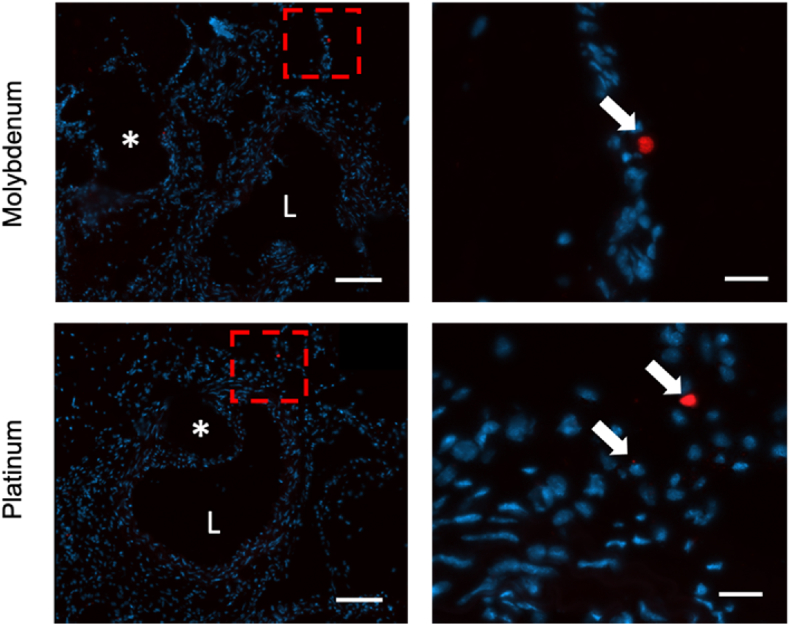
DAPI staining (blue) of mcherry mouse arterial cross sections containing molybdenum (top row) and platinum (bottom row) implants. Macro view of cross sections at 80x normal magnification (left column) and close-up view at 400x normal magnification (right column) are shown. The magnified field is identified by the red dashed box. An “*” identifies the wire implant location and “L” identifies the arterial lumen. Arrows point to mcherry positive macrophages within the tissue that colocalized with DAPI. Images were taken at 400x normal magnification, z-stacked, stitched if necessary, and an extended depth of focus was performed. Scale bar is 100 (left column) and 20 μm (right column). (For interpretation of the references to colour in this figure legend, the reader is referred to the Web version of this article.)

Redlich and colleagues show a cell type sensitive apoptotic effect of Mo on vascular cells. They show *in vitro* that endothelial cells have a higher sensitivity towards apoptotic responses when compared to smooth muscle cells [19]. Although the authors report Mo incubation concentration values that are much higher than what would be seen *in vivo* (0.63 mM vs 0.64 μmol), the trend of endothelial cell sensitivity to molybdenum can be seen in our work; CD31 expression is consistently lower in neointimas surrounding molybdenum wires, while α-SMA expression is unaffected (Fig. 7). Redlich and colleagues also report no significant increase in inflammatory markers generated by monocytes after incubation with varying molybdenum concentrations. We confirm this effect *in vivo* with little to no monocyte derived chronic inflammation surrounding the molybdenum wire at 6 months implantation (Fig. 8). The lack of vascular inflammation at long term implantations of Mo is striking when juxtaposed to the well-known chronic inflammatory responses of Zn and Fe-based degradable materials [45,64,65].

**Fig. 9:**
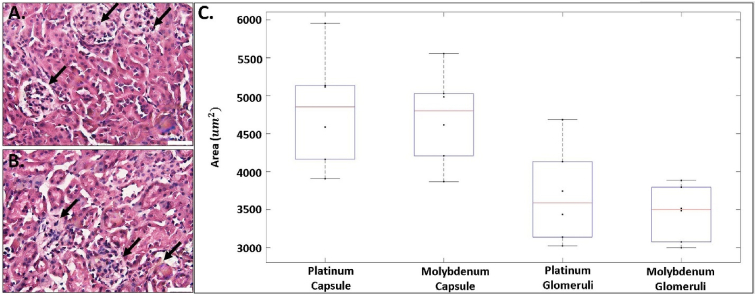
Representative images of H&E-stained kidney cross sections **A.** Platinum. **B.** Molybdenum kidney sections. **C.** Boxplot of the Bowman's capsules and glomeruli area measurements for platinum and molybdenum. For each box, the central line represents the median and the whiskers the minimum and maximum data points. Scale bar in A and B is 20 μm n = 6. * indicates P < 0.05. Black arrows in A and B identify Bowman's capsules and glomeruli.

## 4 Conclusions

We report for the first time the results of the *in vivo* testing of pure Mo as a bioresorbable stent material. Mo possesses excellent mechanical properties for stent applications in its pure form in terms of YS, UTS, and fracture elongation. Our six-month evaluation of pure Mo wire implanted in murine aortas indicate that the critical aspects of biocorrosion, including rate of penetration and the immediate effects of generated products, satisfy the requirements for stent use. The protective film formed on Mo wires is largely uniform and compact and is comprised mainly of Ca, Mo, P, and O, which are benign local byproducts. Decades of research have shown that Mo displays a bioactive chemistry, serving as a cofactor for key enzymes. Indeed, deficits of Mo have been found to be harmful to biological life. Mo implants in mouse arteries avoided cellular responses that contribute to restenosis in terms of SMC hyperplasia, inflammation, and endothelialization of the neointimal tissue. We found a qualitatively pathological remodeling of kidney Bowman's capsule and glomeruli, which could be related to Mo toxicity, although quantification of these changes were not significant. Together, the results suggest that the products of Mo corrosion may exert beneficial or inert effects on the activities of inflammatory and arterial cells locally to the implant. The potentially toxic systemic effect in the kidneys requires further investigations.

If potential toxic effects of Mo can be satisfactorily addressed in future studies, the use of Mo as a bioresorbable stent is very promising. Unlike Zn alloys, due to the high melting temperature of Mo (2623 °C), no mechanical instability originating from temperature sensitivity occurs, which makes Mo a strongly creep-fatigue resistant metal. The markedly high tensile strength of Mo allows the design of a stent with significantly thinner struts (below 70 μm) and therefore improved flexibility and access to narrower vessels. More importantly, the risk of late adverse reactions such as thrombosis (which contributed crucially to the negative outcomes for absorbable polymeric stents) can be reduced with the use of thinner struts [11]. It is worth highlighting that compromise between strength and ductility of Mo implants can be easily achieved by modulating the grain size, without altering its chemical composition, through alloying [20,22].

## CRediT authorship contribution statement

**Malgorzata Sikora-Jasinska:** Writing – original draft, Investigation, Methodology. **Lea M. Morath:** Investigation, Formal analysis. **Maria P. Kwesiga:** Investigation, Formal analysis. **Margaret E. Plank:** Investigation, Formal analysis. **Alexia L. Nelson:** Investigation, Formal analysis. **Alexander A. Oliver:** Investigation, Formal analysis. **Martin L. Bocks:** Writing – review & editing. **Roger J. Guillory:** Supervision, Writing – review & editing. **Jeremy Goldman:** Conceptualization, Supervision, Resources, Project administration, Funding acquisition, Writing – review & editing.

## Declaration of competing interest

The authors have no conflicts to report.
